# Faster DunedinPACE, an epigenetic clock for pace of biological aging, is associated with accelerated cognitive aging among older adults in the Framingham Heart Study

**DOI:** 10.1002/alz.083616

**Published:** 2025-01-03

**Authors:** Micah J. Savin

**Affiliations:** ^1^ Columbia University, New York, NY USA

## Abstract

**Background:**

Biomarkers that predict the heterogenous nature and late onset of cognitive decline are needed to evaluate geroscience interventions designed to delay or prevent the progression of Alzheimer’s Disease and related dementias (ADRD). The DunedinPACE epigenetic clock is designed to measure pace of biological aging, and is therefore a promising candidate biomarker. Here we (1) investigate if DunedinPACE is associated with cognitive aging, and (2) the extent to which these associations are independent of education.

**Method:**

We analyzed data from the Framingham Heart Study Offspring Cohort. Our analysis sample included participants with neuropsychological testing data and blood DNA methylation data collected within 14 months of their baseline cognitive assessment (n = 2,296 non‐Hispanic White adults, 46% male; *M* age = 61.6 ± 9.0 [25‐101y]). A series of growth curve models tested the interactive effects of DundeinPACE and education upon baseline and longitudinal changes in global unadjusted T scores. All models included covariates to adjust for cellular composition of blood samples, smoking status, sex, age at baseline (specified as a quadratic term), and date of blood draw.

**Result:**

A faster DunedinPACE was associated with worse global cognition, and steeper decline in cognition over time (*B*s *= ‐.37 ‐ ‐.45; p*s< .001). After adjusting for education, DunedinPACE remained robustly associated with global cognition and steeper decline in cognition over time (*Bs = ‐.33 ‐ ‐38; ps*<.01). In effect modification analyses, higher education buffered the effect of faster DunedinPACE upon global cognition at baseline (*B* = ‐.21; *p*<.01), but did not significantly modify the effect of DunedinPACE upon cognitive decline over time (*p* = .95).

**Conclusion:**

The presented findings support prior work that highlight the role of systemic biological aging in cognitive decline, and further validates the utility of the epigenetic clock, DunedinPACE, as a potential biomarker for decline in cognitive aging. Further validation of DunedinPACE and its clinical utility as a biomarker for geroscience interventions targeting ADRD is warranted. Greater years of education appears to mitigate some of the deleterious effect of biological aging upon cognition. Future studies should critically examine these relationships among nationally representative samples and diverse samples disproportionately impacted by ADRD.

